# Abnormal expression of cortical cell cycle regulators underlying anxiety and depressive-like behavior in mice exposed to chronic stress

**DOI:** 10.3389/fncel.2022.999303

**Published:** 2022-12-08

**Authors:** Ana Paula Mendes-Silva, Thomas Damien Prevot, Mounira Banasr, Etienne Sibille, Breno Satler Diniz

**Affiliations:** ^1^Centre for Addiction and Mental Health (CAMH), Toronto, ON, Canada; ^2^Department of Psychiatry, Faculty of Medicine, University of Toronto, Toronto, ON, Canada; ^3^Department of Pharmacology and Toxicology, University of Toronto, Toronto, ON, Canada; ^4^School of Medicine, Center on Aging, University of Connecticut Health Center, Farmington, CT, United States; ^5^Department of Psychiatry, School of Medicine, University of Connecticut, Farmington, CT, United States

**Keywords:** cell cycle, cyclin-dependent kinase inhibitors, senescence, prefrontal cortex, anxiety- and depression-like behavior, stress-signaling, chronic restraint stress

## Abstract

**Background:**

The cell cycle is a critical mechanism for proper cellular growth, development and viability. The p16^INK4a^ and p21^Waf1/Cip1^ are important regulators of the cell cycle progression in response to internal and external stimuli (e.g., stress). Accumulating evidence indicates that the prefrontal cortex (PFC) is particularly vulnerable to stress, where stress induces, among others, molecular and morphological alterations, reflecting behavioral changes. Here, we investigated if the p16^INK4a^ and p21^Waf1/Cip1^ expression are associated with behavioral outcomes.

**Methods:**

Prefrontal cortex mRNA and protein levels of p16^INK4A^ and p21^Waf1/Cip1^ of mice (six independent groups of C57BL/6J, eight mice/group, 50% female) exposed from 0 to 35 days of chronic restraint stress (CRS) were quantified by qPCR and Western Blot, respectively. Correlation analyses were used to investigate the associations between cyclin-dependent kinase inhibitors (CKIs) expression and anxiety- and depression-like behaviors.

**Results:**

Our results showed that the PFC activated the cell cycle regulation pathways mediated by both CKIs p16^INK4A^ and p21^Waf1/Cip1^ in mice exposed to CRS, with overall decreased mRNA expression and increased protein expression. Moreover, correlation analysis revealed that mRNA and protein levels are statistically significant correlated with anxiety and depressive-like behavior showing a greater effect in males than females.

**Conclusion:**

Our present study extends the existing literature providing evidence that PFC cells respond to chronic stress exposure by overexpressing CKIs. Furthermore, our findings indicated that abnormal expression of p16^INK4A^ and p21^Waf1/Cip1^ may significantly contribute to non-adaptive behavioral responses.

## Introduction

The cell cycle is a succession of coordinated events which lead to cell division, critical for both the development and viability of multicellular organisms (Kumari and Jat, 2021). The transition from one cell cycle stage to another is regulated by several molecules, including cyclins, cyclin-dependent kinases (CDKs), and cyclin-dependent kinases inhibitors (CKIs). CDKs drive the cell cycle through the accumulation of the dephosphorylated form of the retinoblastoma tumor suppressor protein (pRb) (Mitra et al., 1999; Li et al., 2009). The pRb protein holds an important role in the cell cycle, its inactivation by cyclin-dependent kinases 4 and 6 (CDK4 and CDK6, respectively) releases E2F transcription factors and allows the expression of genes that mediate entry of the transition from G1 to S phase (Giacinti and Giordano, 2006). Of the four phases of the cell cycle, G_1_ is a dynamic stage marked by high rates of biosynthesis and responsiveness to extracellular regulatory signals required for the cell to progress toward division (Bertoli et al., 2013). Control of the G_1_ phase is an essential gatekeeper in the rate of cell cycle progression and is negatively regulated by two main families of CKIs: the Ink4 (p16^INK4a^, p15^INK4b^, p18^INK4*c*^, and p19^INK4d^) and Cip/Kip (p21^Waf1/Cip1^, p27^Kip1^, and p57^Kip2^). The CKI p16^INK4A^ binds directly to CDK4 and CDK6, blocking phosphorylation of the pRb and subsequent traversal of the G1/S cell cycle checkpoint. In addition, the CKI p21^Waf1/Cip1^ maintains cell quiescence, blocking progression into S-phase, by inhibiting the cyclin-CDK2, cyclin-CDK1, and cyclin-CDK4, 6 complexes (Georgakilas et al., 2017). If upregulated, p21^Waf1/Cip1^ causes cell growth arrest at the G2 phase and is required for sustained G2 arrest following DNA damage (Bunz et al., 1998). The cell cycle machinery is known predominantly for regulating cellular proliferation, although accumulating evidence indicates that cell cycle proteins may also control cellular senescence and apoptosis, especially in postmitotic cells (Sapieha and Mallette, 2018). Postmitotic cells, also known as terminally differentiated cells, have long been considered to be permanently in G0. However, increasing evidence indicates that postmitotic cells such as neurons can re-enter the cell cycle in response to internal and external stimuli such as stress and this can be critical for their functions (Pajalunga et al., 2007; Sah et al., 2021).

Stress is characterized as a state in which intrinsic or extrinsic stimuli evoke a dynamic and complex repertoire of biological, physiological, and behavioral adaptive responses of the organism (Chrousos and Gold, 1992; Chrousos, 2009; Yaribeygi et al., 2017). Adaptive responses are required to reestablish homeostasis and range from transient molecular changes to cell death based on the stimulus’s type, timing, and severity (Lupien et al., 2009). Several studies have shown that stress can be either a triggering or aggravating factor for many pathological conditions, including cancer (Moreno-Smith et al., 2010), depression (Yang et al., 2015), post-traumatic stress disorder (Sherin and Nemeroff, 2011), and neurodegenerative disorders (Hemmerle et al., 2014; Justice, 2018; Song et al., 2020). Stress response involves a range of well-conserved signaling pathways that depend upon highly interconnected neuroendocrine, cellular, and molecular pathways (Harding et al., 2003; Poon, 2016; Nava et al., 2020). The hypothalamic-pituitary-adrenal axis and the autonomic nervous system are key components of the stress system, and they interact with different brain regions and tissues/organs in the periphery to elicit the cellular and molecular adaptive responses against the stimuli (Miller and O’Callaghan, 2002). To date, it is mechanistically not clarified how senescence status in brain cells is activated by stress and how it is connected to maladaptive behavioral responses. The literature consistently shows that suppression of adult hippocampal neurogenesis caused by stress (Besnard and Sahay, 2016; Drew and Huckleberry, 2017; Huckleberry et al., 2018) might contribute to a maladaptive emotional and stress-coping strategy. Several lines of evidence have indicated that the prefrontal cortex (PFC) is particularly vulnerable to stress, where stress induces volumetric (de Lange et al., 2008; McEwen et al., 2016; Misquitta et al., 2021), cytoarchitectural (Misquitta et al., 2021), and morphological changes, including the reduction of dendrite length, branching, and spine density (McEwen, 1997; McEwen and Magarinos, 1997; Radley et al., 2006; Arnsten, 2009; Kang et al., 2012; Arnsten et al., 2015; Qiao et al., 2016; Moreno et al., 2017; Banasr et al., 2021; Bernardo et al., 2022). In addition, altered PFC neuronal activity is associated with social defeat-induced depression- and anxiety-like behavior in mice, and learning and memory impairments (Sullivan and Gratton, 2002; Lupien et al., 2009; Felix-Ortiz et al., 2016; Burgos-Robles et al., 2017; Planchez et al., 2019).

Previous studies have shown that p16^INK4a^ and p21^*Waf*1/Cip1^ play critical roles in cell cycle arrest in neurons in the hippocampus by preserving cellular integrity and avoiding cell death (Sapieha and Mallette, 2018; Sah et al., 2021). Building on observations that p16^INK4a^ and p21^Waf1/Cip1^ expression in the brain is sustained postnatally, Le et al. (2018) have shown that p16^INK4a^ expression mediates the loss of neurogenesis in the hippocampus of mice exposed to ionizing radiation. In a recent report, p16^INK4a^ appears to prevent a neural stem cell release from quiescence when neurogenic stimuli are present, suggesting that p16^*INK*4a^ is crucial to the self-renewal capacity during aging (Micheli et al., 2019). Moreover, Pechnick et al. (2011) showed that proliferation of adult neurons in the hippocampus is restrained by CKIs and loss of p21^Waf1/Cip1^ function results in the release of progenitors from cell cycle block. Although the cellular bases of senescence underlying the stress response have been extensively investigated in the hippocampus, the effects of cell cycle arrest in PFC remain unknown.

In order to investigate whether the cell cycle regulators are involved in behavioral changes, we first measure the mRNA and protein levels of CKIs p16^INK4a^ and p21^*Waf*1/Cip1^ in the PFC of mice exposed to chronic restraint stress (CRS). Then we assessed whether changes in p16^INK4a^ and p21^Waf1/^mRNA and protein levels ^Cip1^ were associated with anxiety- and depressive-like behaviors. Our findings open new avenues for cell cycle-related research and provide evidence that postmitotic cells have a complex stress response involving CKIs, extending our understanding of cellular senescence underlying depressive symptoms.

## Materials and methods

### Animals

Mouse brain samples were obtained from the chronic stress mouse brain bank of Drs. Prevot and Banasr. Behavioral outcomes induced by CRS are described in Prevot et al. (2021). All procedures followed guidelines set by the Canadian Council on Animal Care (CCAC) and were approved by CAMH Animal Care Committee. Briefly, 8-week-old C57BL/6J mice (Jackson Laboratory, Bar Harbor, ME, USA; *n* = 48, eight animals/group, 50% females) were housed under standard conditions with a 12 h light/dark cycle and provided with *ad libitum* access to food and water. Animals were subjected to either 0, or 3, 14, 21, 28, or 35 days of CRS. Each restraint stress session consists of placing mice in a 50 ml Falcon^®^ tube (Thermo Fisher Scientific, Waltham, MA, USA) perforated on each end to allow air circulation. Restraint sessions occurred twice daily for 1 h during the light cycle (7:00 a.m.–7:00 p.m.), separated by a minimum of 2 h. CRS-exposed animals were single-housed throughout the stress exposure, and control animals were group-housed until behavioral assessment.

### Behavioral tests

Behavioral outcomes were assessed at each time point, and the methods and results were presented in Prevot et al. (2021). Animals were tested in the sucrose consumption test and the *Phenotyper*™ *test*. Each animal coat degradation and weight were also measured.

#### Sucrose consumption test

Anhedonia was assessed on each time point of the CRS protocol using a sucrose consumption test. The test consists of habituation to sucrose (1%) for 48 h and presentation of pre-weighted drinking bottles for 1 h after 16 h of fluid deprivation. Water consumption was measured on a consecutive day following the same procedure. Sucrose preference was calculated according to the formula: sucrose preference = (sucrose intake)/(sucrose intake + water intake) × 100.

#### *PhenoTyper*™ *test*

The *PhenoTyper*™ *test* was performed according to a previous publication (Prevot et al., 2019) on each time point. Residual avoidance (RA) was calculated for each mouse based on the time spent in food (RA FZ) and shelter (RA SZ) zones after the light challenge, as previous described (2019).

#### Physiological changes

Animals’ coat state quality was scored across seven anatomical areas 0, 0.5, or 1, from maintained to unkempt, and a sum was calculated. Weight was tracked weekly and expressed as percent change (%) to measure deviation from normal development.

### Protein and RNA extraction

After completing each time point of CRS protocol, all animals were euthanized, the brain was removed, and the PFC was dissected, collected, and separated into 1.5 ml Eppendorf tubes (Eppendorf, UK) and then rapidly shock-frozen on dry ice and stored at –80°C until used. Protein and RNA were extracted from the samples using the AllPrep RNA/Protein Kit (Qiagen, Hilden, Germany) and stored at –80°C.

### Western blot

Total protein concentration was determined using a BCA protein assay. Protein homogenates (30 μg) were loaded and separated by sodium dodecyl sulfate-polyacrylamide gel electrophoresis (SDS-PAGE). The membranes were incubated overnight with primary antibodies against p16^INK4a^ (rabbit polyclonal antibody ab189034, Abcam, Cambridge, MA; 1:1,000), p21^Waf1/Cip1^ (mouse monoclonal antibody sc-6246, Santa Cruz Biotechnology, Santa Cruz, CA; 1:1,000), GAPDH (rabbit polyclonal antibody E-AB-20072, Elabscience Biotechnology, Wuhan, China; 1:2000), and then incubated with the related secondary antibodies at a 1:10,000 dilution. The GAPDH levels were used to normalize the levels of the targeted proteins quantified by Western blot.

### Real-time reverse transcription-polymerase chain reaction

Extracted RNA was converted into cDNA by reverse transcription (Kadakkuzha et al., 2013). Briefly, 1 μg of RNA was used with SuperScript VILO cDNA Synthesis Kit (Thermo Fisher, Waltham, Massachusetts, EUAUSA) according to the manufacturer’s instructions. Quantitative real-time reverse transcription-polymerase chain reaction (RT-PCR) was performed using gene-specific primers for p16^INK4a^ (Forward 5′-CCGAACTCTTTCGGTCGTAC-3′; Reverse 5′-AGTTCGAATCTGCACCGTAGT-3′; amplicon size 94 bp), p21^Waf1/Cip1^ (Forward 5′-TGTCGCTGTCTT GCACTCTG-3′; Reverse 5′-GACCAATCTGCGCTTGGAGT-3′; amplicon size 128 bp) and GAPDH (Forward 5′-AACTCCCACTCTTCCACCT-3′; Reverse 5′-CACCACC CTGTTGCTGTA-3′; amplicon size 111 bp) and SYBR Green PCR master mix (Applied Biosystems Carlsbad, CA, USA) for detection in Bio-rad CFX96 Touch PCR Detection System (Bio-Rad Laboratories, Hercules, CA, USA). All the qPCR amplifications were performed in triplicate in a total volume of 20 μl containing 4 μl of H2O, 3 μl of cDNA, 10 μl of 2X Master Mix, 1.5 μl of 10 μm (each) forward and reverse primers. Data were analyzed by standard ΔCq method (2^–Δ^ ^Δ^ ^Cq^) where ΔCq is the difference between the genes of interest and GAPDH control Cq values for each sample (Livak and Schmittgen, 2001).

### Statistical analysis

Data analysis was performed using SPSS software (IBSM SPSS statistic 24). *A priori*, we evaluated the normal distribution of the data. Data following normal distribution were analyzed using one-way ANOVA followed by Dunnett’s *post hoc* test to compare the impact of CRS exposure (control vs. each CRS group) on p16^INK4a^ and p21^Waf1/Cip1^ expression. Moreover, data with a non-normal distribution were analyzed using a non-parametric Kruskal–Wallis test, followed by Bonferroni/Dunn *post hoc* tests to compare the impact of CRS exposure (control vs. each CRS group) on p16^INK4a^ and p21^Waf1/Cip1^ expression. Analysis of covariance (ANCOVA) was applied to investigate the influence of sex on p16^INK4a^ and p21^Waf1/Cip1^ expression and anxiety- and depression-like behaviors. Z-scores were calculated to assess consistency of behavioral phenotypes across tests, referred to as z-emotionality using averaged z-scores of behavioral tests as described previously (Guilloux et al., 2011). We carried out Pearson’s or Spearman’s correlation analysis when adequate to investigate the association between an overall expression of mRNA and protein of p16^INK4a^ and p21^Waf1/Cip1^, and anxiety- and depression-like behaviors. Significance was set at a *p*-value ≤ 0.05.

## Results

### Behavioral outcomes

Full report of the behavioral outcomes was presented in Prevot et al. (2021). In summary, Prevot et al. (2021) showed that shorter durations of chronic stress exposure induced anxiety-like behaviors (within 7 days), while the longer duration induced anxiety- and anhedonia-like behaviors (35 days). The anxiety- and anhedonia-like behaviors were reflected by increased shelter zone time and decreased food zone time, lower sucrose consumption, lower weight gain, and worse coat state.

### Abnormal cyclin-dependent kinase inhibitors expression in the prefrontal cortex of mice under chronic restraint stress

#### p16^INK4a^ and p21^Waf1/Cip1^ mRNA expression

**FIGURE 1:**
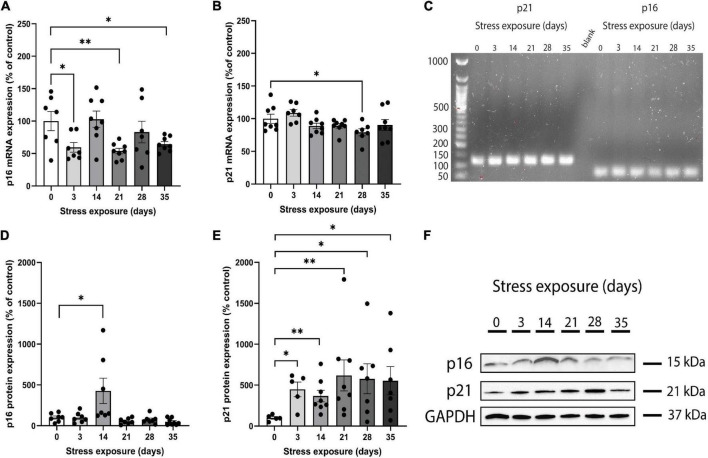
Chronic restraint stress (CRS) induces abnormal mRNA and protein expression of p16^INK4a^ and p21^Waf1/Cip1^ in mice. **(A)** The p16^INK4a^ mRNA expression levels were lower in mice submitted to CRS than the control group after 3, 21, and 35 days of stress exposure. **(B)** The p21^Waf1/Cip1^ mRNA expression levels were lower in mice submitted to CRS compared to the control group after 28 days of stress exposure. **(C)** Full length electrophoresis gel image showing single amplicon bands after RT-qPCR for p21 and p16^INK4a^ genes expressed in PFC of mice submitted to CRS (0–35 days of exposure). **(D)** The p16^INK4a^ protein expression levels were higher in mice submitted to CRS compared to the control group after 14 days of stress exposure. **(E)** The p21^Waf1/Cip1^ protein expression levels were higher in mice submitted to CRS compared to the control group after 3, 14, 21, 28, and 35 days of stress exposure. **(F)** Western blot analysis showing the protein expression levels of p16^INK4a^, p21, and GAPDH in PFC of mice submitted to CRS (0–35 days of exposure). Data showed were obtained in both male and female, and were presented as mean ± SEM. *p < 0.05, ^**^p < 0.01.

Real-time qPCR was used to measure p16^INK4a^ and p21^Waf1/Cip1^ mRNA expression in PFC samples obtained from the whole cohort and the size and specificity of each amplicon was confirmed by agarose gel electrophoresis (Figure 1C) and melting curve (Supplementary Figure 4). Analysis of variance showed that C57BL/6J mice PFC presented a significant main effect of stress on the p16^INK4a^ mRNA expression across groups (*F*_(5,39)_ = 3.907, *p* = 0.006). Repeated-measures ANCOVA of p16^INK4a^ mRNA levels revealed that the significant main effect of CRS (*F*_(11,33)_ = 2.509, *p* = 0.02) remained after controlling for sex. The ANCOVA model also showed that there were no main effects of both sex (*F*_(1,33)_ = 1.895, *p* = 0.178) and stress*sex interaction (*F*_(5,33)_ = 1.147, *p* = 0.355). Pairwise comparisons of CRS groups versus control showed that p16^INK4a^ mRNA expression reduced significantly on days 3 (*p* = 0.031), 21 (*p* = 0.007), and 35 (*p* = 0.029; Figure 1A). In addition, there were no significant differences in p16^INK4a^ mRNA expression on days 14 (*p* = 0.877) and 28 (*p* = 0.466) when compared to the control group (Figure 1A).

Results of ANOVA analysis showed that p21^Waf1/Cip1^ mRNA expression was different among groups (*F*_(5,40)_ = 2.924, *p* = 0.024). When sex was added as a co-factor, the difference in the p21^Waf1/Cip1^ mRNA expression across groups did not remain statistically significant (*F*_(11,34)_ = 1.475, *p* = 0.186). In addition, we found no main effect of both sex (*F*_(1,34)_ = 0.044, *p* = 0.836) and stress*sex interaction (*F*_(5,34)_ = 0.545, *p* = 0.741) on p21^Waf1/Cip1^ mRNA expression. Pairwise analysis of CRS groups versus control showed that p21^Waf1/Cip1^ mRNA expression reduced significantly on day 28 (*p* = 0.037; Figure 1B). There were no significant differences in p21^Waf1/Cip1^ mRNA expression on days 3 (*p* = 0.330), 14 (*p* = 0.194), 21 (*p* = 0.180), and 35 (*p* = 0.387) when compared to the control group (Figure 1B).

#### p16^INK4a^ and p21^Waf1/Cip1^ protein expression

Western blot was performed on PFC samples obtained from the whole cohort (Figure 1F). Kruskal-Wallis test revealed that p16^INK4a^ protein expression was different among groups [*X*^2^_(5,42)_ = 19.003, *p* = 0.002]. After including sex as a co-factor, the difference in the protein expression of p16^INK4a^ across groups remained statistically significant [*F*_(11,30)_ = 5.043, *p* = 0.0001]. The ANCOVA model revealed a significant main effect of stress*sex interaction [*F*_(5,30)_ = 3.665, *p* = 0.010], and no main effect of sex [*F*_(1,30)_ = 2.164, *p* = 0.152]. Pairwise analysis showed that mice submitted to 14 days of CRS increased significantly p16^INK4a^ protein levels (*p* = 0.025) compared to control group (Figure 1D). There were no significant differences in p16^INK4a^ protein expression on days 3 (*p* = 0.749), 21 (*p* = 0.355), 28 (*p* = 0.338), and 35 (*p* = 0.083) when compared to the control group (Figure 1D).

We found a marginally significant difference on p21^Waf1/Cip1^ protein expression among groups [*X*^2^_(5,39)_ = 10.790, *p* = 0.056]. When sex was considered as a covariate, there was no significant difference in the p21^Waf1/Cip1^ protein expression across groups [*F*_(11,33)_ = 2.066, *p* = 0.061], but a main effect of sex [*F*_(1,33)_ = 4.518, *p* = 0.043] was found. In addition, there was no main effect of stress × sex interaction [*F*_(5,33)_ = 1.294, *p* = 0.296]. Pairwise contrast analysis were performed and results showed that p21^Waf1/Cip1^ protein expression increased significantly on days 3 (*p* = 0.016), 14 (*p* = 0.005), 21 (*p* = 0.005), 28 (*p* = 0.045), and 35 (*p* = 0.028) when compared to the control group (Figure 1E).

#### Cortical cyclin-dependent kinase inhibitors expression and behavior changes

##### Cyclin-dependent kinase inhibitors expression is associated with anxiety and depressive-like behavior

**FIGURE 2:**
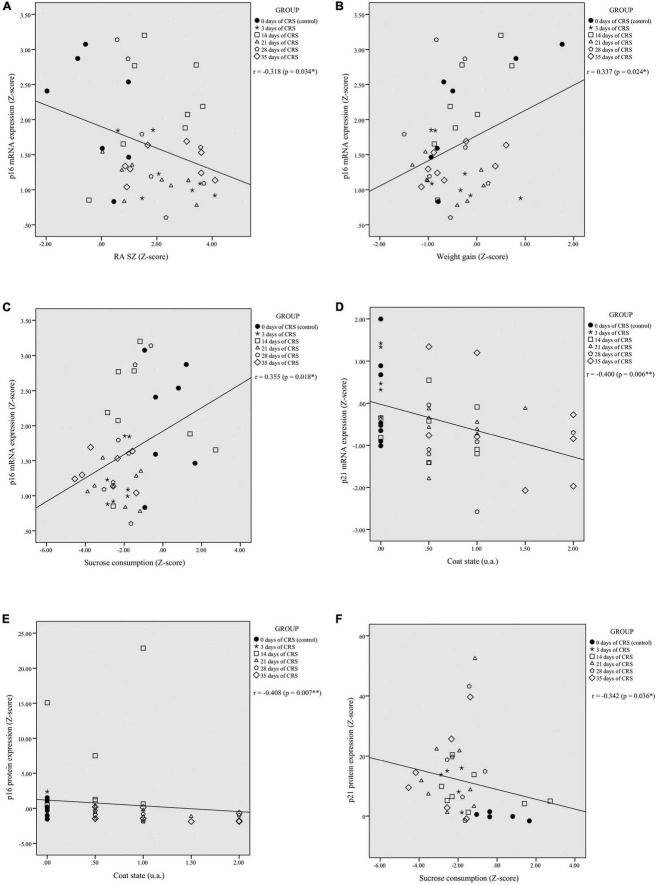
Chronic restraint stress (CRS)-induced alterations in PFC p16^INK4a^ and p21^Waf1/Cip1^ mRNA and protein expression levels correlates with behavioral performances. The p16^INK4a^ mRNA expression levels were significantly correlated with residual avoidance in shelter zone (RA SZ) **(A)**, weight gain **(B)**, and sucrose consumption **(C)**. The p21^Waf1/Cip1^ mRNA levels showed a significant negative correlation with coat state **(D)**. The p16^INK4a^ protein expression levels were negatively correlated with coat state **(E)** and the p21^Waf1/Cip1^ protein expression levels were negatively correlated with sucrose consumption **(F)**. *p < 0.05, **p < 0.01.

Correlation analyses were performed to assess potential relationships between PFC mRNA and protein expression of p16^INK4a^ and p21^Waf1/Cip1^ and behavioral outcomes. The p16^INK4a^ mRNA expression was correlated negatively with RA in shelter zone (*r* = −0.318, *p* = 0.034; Figure 2A), correlated positively with weight gain (*r* = 0.337, *p* = 0.024; Figure 2B), and sucrose consumption (*r* = 0.355, *p* = 0.018; Figure 2C). There were no significant correlations with RA in food zone (*r* = −0.251, *p* = 0.097) and coat state (*r* = −0.164, *p* = 0.280; Supplementary Figures 2A,B, respectively). The p21^Waf1/Cip1^ mRNA expression was correlated negatively with coat state (*r* = −0.400; *p* = 0.006; Figure 2D), but not showed a significant correlation with RA in shelter (*r* = −0.009; *p* = 0.952) and food zones (*r* = −0.068; *p* = 0.651), weight gain (*r* = −0.223; *p* = 0.136), or sucrose consumption (*r* = 0.094; *p* = 0.539; Supplementary Figures 2C–F, respectively).

The p16^INK4a^ protein levels were correlated negatively with coat state (*r* = −0.408, *p* = 0.007; Figure 2E), but showed no significant correlation with RA in food (*r* = −0.066, *p* = 0.679) and shelter zones (*r* = −0.003, *p* = 0.985), weight gain (*r* = 0.209, *p* = 0.185), or sucrose consumption (*r* = 0.217, *p* = 0.173; Supplementary Figures 2G–J, respectively). The p21^Waf1/Cip1^ protein expression was correlated negatively with sucrose consumption (*r* = −0.342, *p* = 0.036; Figure 2F), but showed no significant correlation with RA in food (*r* = 0.128, *p* = 0.436) and shelter zones (*r* = −0.063, *p* = 0.705), weight gain (*r* = −0.160, *p* = 0.332), or coat state (*r* = 0.250, *p* = 0.124; Supplementary Figures 2K–N, respectively).

**FIGURE 3:**
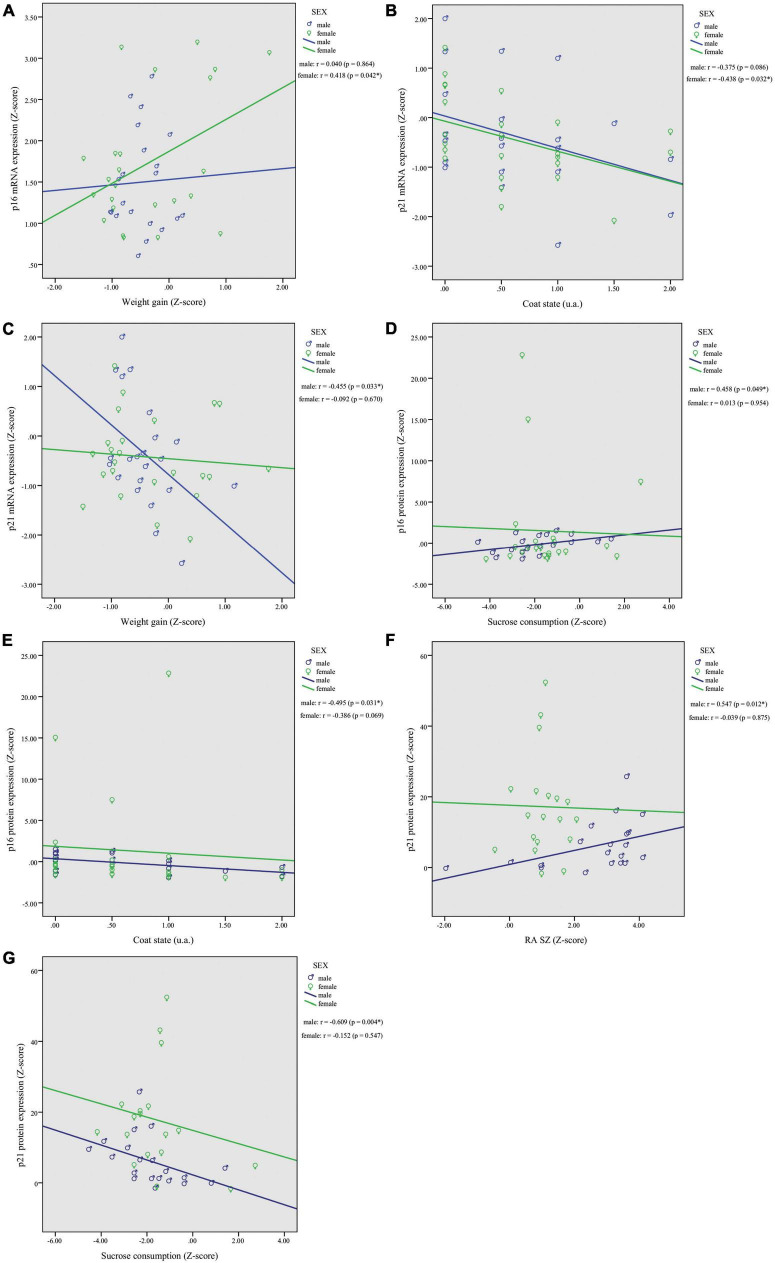
Chronic restraint stress (CRS) leads to sexually dimorphic p16^INK4a^ and p21^Waf1/Cip1^ mRNA and protein expression changes in the PFC with associated behavioral responses. In females, correlations analysis revealed a significant association of p16^INK4a^ mRNA expression with weight gain **(A)**, and p21^Waf1/Cip1^ with worsened coat state **(B)**. In males, we found a significant correlation of p21^Waf1/Cip1^ mRNA expression and weight gain **(C)**. Higher protein levels of p16^INK4a^ showed a positive association with sucrose consumption **(D)** and worsened coat state **(E)**, and higher protein levels of p21^Waf1/Cip1^ were associated with greater (RA SZ) **(F)** and reduced sucrose consumption **(G)**. Data showed were obtained in both male and female. *p < 0.05, **p < 0.01.

Based on the observed mRNA and protein expression associations with behavioral outcomes, we decided to split the dataset by sex to investigate potential sexual dimorphism effects. In females, correlations analysis revealed a significant association of p16^INK4a^ mRNA expression with weight gain (*r* = 0.418, *p* = 0.042; Figure 3A), and p21^Waf1/Cip1^ with worsened coat state (*r* = −0.438, *p* = 0.032; Figure 3B). In males, correlation analysis showed a significant association of p21^Waf1/Cip1^ mRNA expression and weight gain (*r* = −0.455, *p* = 0.033; Figure 3C). Moreover, p16^INK4a^ and p21^Waf1/Cip1^ protein expression were correlated with behavior exclusively in males. The p16^INK4a^ protein levels were correlated positively with sucrose consumption (*r* = 0.458, *p* = 0.049; Figure 3D) and negatively with coat state (*r* = −0.495, *p* = 0.031; Figure 3E). In addition, p21^Waf1/Cip1^ protein levels were correlated positively with RA in shelter zone (*r* = 0.547, *p* = 0.012; Figure 3F) and negatively with sucrose consumption (*r* = −0.609, *p* = 0.004; Figure 3G). No other correlations reached statistical significance in females or males; results are shown in Supplementary Figure 3.

Finally, we did not find a significant correlation between mRNA and protein expression levels of p16^INK4a^ (*r* = 0.208, *p* = 0.192) and p21^Waf1/Cip1^ (*r* = −0.113, *p* = 0.494).

## Discussion

In this exploratory study, we used CRS as a tool to investigate the influence of cell cycle regulators (p16^INK4a^ and p21^Waf1/Cip1^) expression on mice behavior. Our results demonstrated that mice exposed to CRS showed a decreased mRNA expression of p16^INK4a^ (within 3, 21, and 35 days) and p21^Waf1/Cip1^ (within 21 days), but increased protein levels of p16^INK4a^ (within 14 days) and p21^Waf1/Cip1^ (within 3, 14, 21, 28, and 35 days) in the PFC. Further, the PFC mRNA and protein expression of p16^INK4a^ and p21^Waf1/Cip1^ were correlated with anxiety- and depressive-like behaviors showing a greater effect in males than females. To best of our knowledge, this is the first study to revealed that p16^INK4a^ and p21^Waf1/Cip1^ are involved in the PFC response to chronic stress and their expression are significantly associated with behavioral performances.

Accumulating evidence indicates that cellular responses to exposure to different stimuli are largely adaptive by means of a complex protein network responsible for the recovery and maintenance of genetic integrity (Bartek and Lukas, 2007; Jackson and Bartek, 2009; Poon, 2016). However, if the damage caused by the stimuli such as stress cannot be repaired, the cell will commit to two primary fates: senescence, or apoptosis (Poon, 2016; Nava et al., 2020). Our results suggested a differential changes in PFC mRNA and protein expression of p16^INK4a^ and p21^Waf1/Cip1^ during stress exposure. The mRNA expression seems to be inhibited by stress while is observed overexpression of protein with longer exposure to CRS. Assuming that mRNA and protein abundance is regulated through a dynamic balance between their synthesis, degradation rates, and mean lifetime, a direct correlation between the amounts of an mRNA and its corresponding protein might not be observed (Maier et al., 2009; Hoernes et al., 2016). Taking together these results may suggest a cooperative stress response eliciting two distinct damage-response pathways responsible for (1) inhibit transcription, avoiding increase the DNA damage, and (2) cell cycle arrest by overexpression of p16^INK4a^ and p21C^IP1/WAF1^ proteins. Evidence suggests that inhibition of DNA synthesis may prevent the accumulation of DNA damage and worse consequences such as apoptosis (Kim and Jinks-Robertson, 2012). Moreover, increased levels of p16^INK4a^ and p21^Waf1/Cip1^ proteins may suggest a secondary activation of the Rb protein phosphorylation which also inhibits the DNA synthesis and triggers the cell to arrest in G1 (Kim and Jinks-Robertson, 2012). Kfoury et al. (2018) showed that mRNA and protein of p16^INK4a^ and p21^Waf1/Cip1^ dynamically changed and presented a cooperative phenomenon that is necessary to protect astrocytes against an aberrant proliferation and DNA mutations.

The transition from acute senescence state to a permanent one typically involves prolonged CDK–cyclin activity inhibition by increased CKIs expression. In line with results published by Purvis et al. (2012), we observed an increased protein levels of p21^Waf1/Cip1^ and transient increased p16^Ink4a^ protein levels over CRS exposure. Several findings of a senescent-like phenotype postmitotic neurons and glia in mice and human indicate that this mechanism might not be exclusive to proliferating cells (Sedelnikova et al., 2004; Jurk et al., 2012; Piechota et al., 2016; Fielder et al., 2017). A recent study showed that both p21^WAF1/CIP1^ and p16^INK4a^ were expressed by astrocyte and induced senescence state in early stages of the and amyotrophic lateral sclerosis, whereas p21^WAF1/CIP1^ was exclusively expressed by neurons and might reflect a more general mechanism of age-related cell-cycle dysregulation (Vazquez-Villaseñor et al., 2020). Le et al. (2018) found that the absence of p16^INK4a^ mRNA expression could to some extent restore neurogenesis in the dentate gyrus following exposure to ionizing radiation. In astrocytes and microglia, senescence seems to contribute to the disruption of the glia–neuron interaction, glial loss and neuronal atrophy and may underlie, in part, the development of age-related brain pathologies such as Alzheimer’s disease and depression (Cotter et al., 2001; Drevets, 2001; Rajkowska and Miguel-Hidalgo, 2007; Banasr et al., 2010; Mombach et al., 2015; Crowe et al., 2016; Safaiyan et al., 2016; Christman et al., 2020). Cellular senescence might be a key mechanism in which cells temporarily lose their replicative phenotype as an attempt to respond to stress and reverse or compensate for decreased cell number and loss of neuroplasticity (Sikora et al., 2021). However, given that the primary consequence of senescence activation in postmitotic cells are still not completely understood.

Combining our findings, the correlation analysis demonstrated that lower p16^INK4a^/p21^CIP1/WAF1^ mRNA levels are associated with greater anxiety, and anhedonia deficits, whereas higher p16^INK4a^/p21^CIP1/WAF1^ protein levels are associated with greater coat deterioration and reduced sucrose consumption. The mechanisms underlying how stress affects body weight and food intake are not well understood. Weight gain or lack thereof in CRS animals was associated with chronic elevation of corticosterone level and HPA axis dysregulation without change in food intake (Jeong et al., 2013). It is possible that the significant association between p16^INK4a^/p21^CIP1/WAF1^ expression and weight gain is linked to this mechanism. Different studies will need to be designed to specifically address this question. Although the main focus of this study was investigating the effects of PFC CKI’s expression in behavior we included an equal number of male and female mice to account for sex. In CRS female mice, p16^INK4a^ mRNA levels correlated positively with weight gain and p21^Waf1/Cip1^ mRNA expression levels correlated with worsened coat state. In CRS male mice, p21^CIP1/WAF1^ mRNA expression levels correlated negatively with weight gain, p16^INK4a^ protein expression levels correlated positively with greater sucrose consumption and correlated negatively with coat state, and p21^Waf1/Cip1^ protein expression levels correlated positively with RA SZ and correlated negatively with sucrose consumption. The literature suggests that females are more affected by stress and exhibit more symptoms of stress-related disorders (Giovanniello et al., 2021; Woodburn et al., 2021), although we were unable to find significant correlations between CKI’s protein levels and behavioral outcomes in females. This could be due to variability in the estrus cycle which we did not monitor in this study or to potential sexual dimorphism. Indeed, in prior studies, sexual dimorphism in cell cycle regulation and DNA repair by p16^INK4a^ and p21^CIP1/WAF1^ was shown to have a crucial role in cancer incidence such as glioblastoma (Ostrom et al., 2016; Kfoury et al., 2018). Future studies specifically designed for studying sexual dimorphism and defining the cellular effects of an abnormal CKI’s expression in PFC would be needed to answer this question.

This study has several limitations. First, the relatively small number of animals per group and per sex, the cross-sectional design also limit the interpretation of our findings regarding the progression of the response to chronic stress. Additionally, the study design did not allow for drawing any causal link between cell cycle regulators’ expression and depression-like behavior. We will need to investigate further to understand the underlying mechanism of time-dependent effects of stress on cortical p16^INK4a^ and p21^CIP1/WAF1^ expression, as well as its potential influence on a difference in cell cycle response between males and females. Although cognitive deficits are part of depressive symptoms and could have brought additional information related to PFC changes, we chose not to perform any cognitive tasks driven by feasibility concerns with a high volume of animals. Further studies will be necessary to investigate the effects of p16^INK4a^ and p21^CIP1/WAF1^ expression changes on cognitive functions and memory. Finally, we also did not measure p16^INK4a^ and p21^CIP1/WAF1^ levels in specific brain cells or other brain regions. Future studies should investigate the induction of senescence through p16^INK4a^ and p21^Waf1/Cip1^ in human brain tissue from patients with stress-related disorders to confirm the translation of our findings.

Despite these limitations, the present exploratory study extends the existing literature providing evidence that postmitotic cells have a complex stress response involving senescence markers, and. suggests a link between p16^INK4a^ and p21^Waf1/Cip1^ expression and anxiety- and depressive-like behaviors in mice submitted to CRS.

## Data availability statement

The original contributions presented in this study are included in the article/Supplementary material, further inquiries can be directed to the corresponding author.

## Ethics statement

This animal study was reviewed and approved by the CAMH Animal Care Committee.

## Author contributions

AM-S conceptualized the study, conducted all the wet lab and statistical analyses, interpretation of results, and wrote the original manuscript draft. TP conceptualized the study, performed the behavioral tests, and contributed to the original manuscript draft. MB and ES contributed to the manuscript reviewing, editing, and providing critical intellectual input about the analysis and results. BD developed the study concept, oversaw all analyses, and all stages of the manuscript preparation. All authors reviewed and approved the manuscript before submission.
