# Supplementary material for: Abnormal expression of cortical cell cycle regulators underlying anxiety and depressive-like behavior in mice exposed to chronic stress
Source: Front Cell Neurosci. 2022 Dec 8;16:999303. doi: 10.3389/fncel.2022.999303 (PMC9772437; doi:10.3389/fncel.2022.999303)

Supplementary Material

1 Supplementary Figures

1.1 Supplementary Figure 1 - Chronic restraint stress (CRS) induces changes in cortical p16^{INK4a} and p21$^{Waf1/Cip1}$ mRNA and protein expressions and behavioral performances. a)

Regulation of cell cycle by intrinsic cooperation between p16^{INK4a} and p21$^{Waf1/Cip1}$ in response to stress may generates a senescence state in PFC cells. **b)** The deregulation of the cell cycle in PFC cells may resulted in worse behavioral performances such as higher residual avoidance in food and shelter zones, lower sucrose consumption, lower weight gain and worse coat state. **c)** Overall changes in cortical p16^{INK4a} and p21$^{Waf1/Cip1}$ mRNA and protein expressions and behavioral responses across stress exposure groups (0-35 days of CRS). **d)** p16^{INK4a} and p21$^{Waf1/Cip1}$ expression might affect behavioral performances.

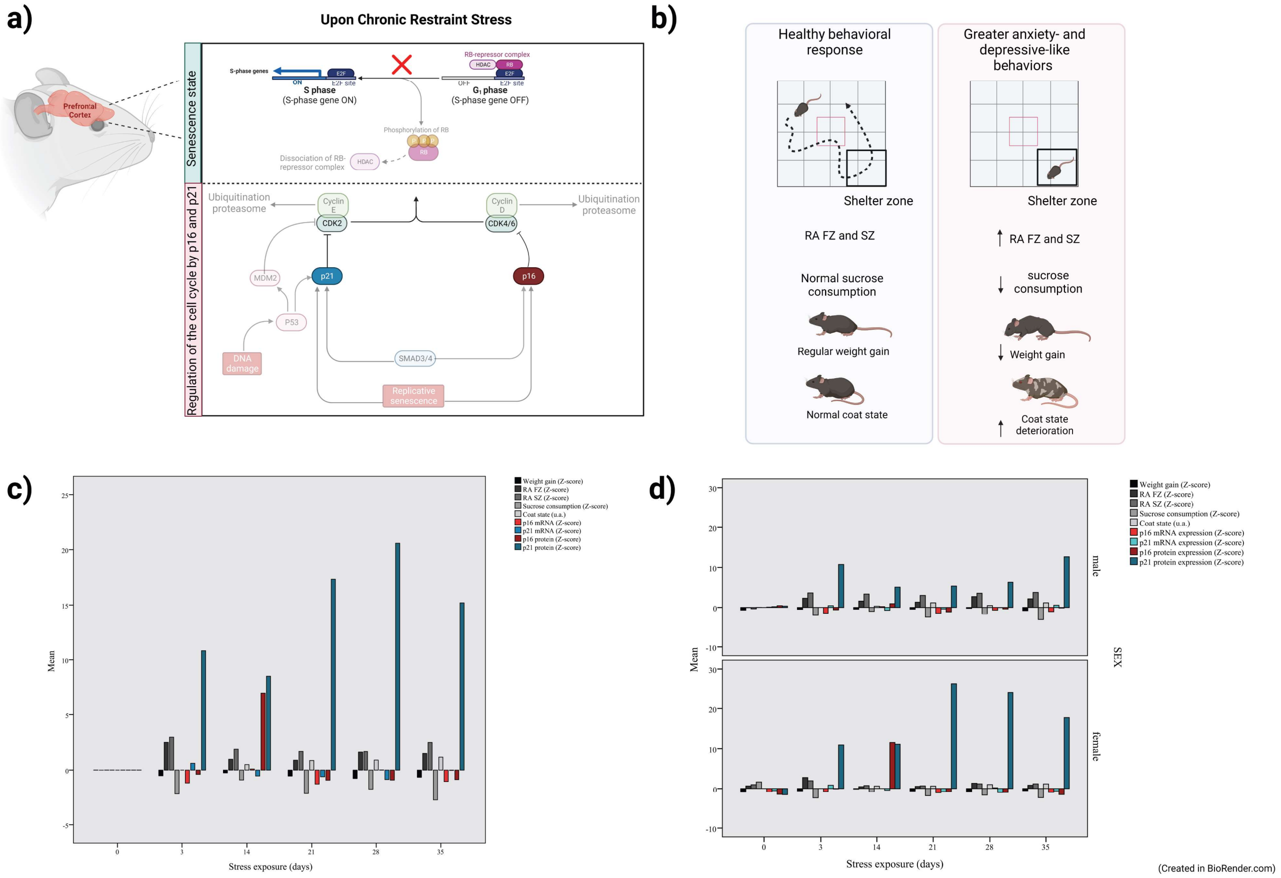

1.2 Supplementary Figure 2 - Non-significant correlations of cortical $p16^{INK4a}$ and $p21^{Waf1/Cip1}$ mRNA and protein expression levels and behavioral performances in in mice submitted to Chronic restraint stress (CRS). The $p16^{INK4a}$ mRNA expression levels were not significantly correlated with residual avoidance in food zone (RA FZ) **(a)** and coat state **(b)**. The $p21^{Waf1/Cip1}$ mRNA expression levels were not significantly correlated with residual avoidance in food zone (RA FZ) **(c)**, residual avoidance in shelter zone (RA SZ) **(d)**, weight gain **(e)** and sucrose consumption **(f)**. Moreover, the $p16^{INK4a}$ protein expression levels were not significantly correlated with residual avoidance in food zone (RA FZ) **(g)**, residual avoidance in shelter zone (RA SZ) **(h)**, weight gain **(i)**, and sucrose consumption **(j)**. The $p21^{Waf1/Cip1}$ protein levels were not correlated with residual avoidance in food zone (RA FZ) **(k)**, residual avoidance in shelter zone (RA SZ) **(l)**, weight gain **(m)** and coat state **(n)**.

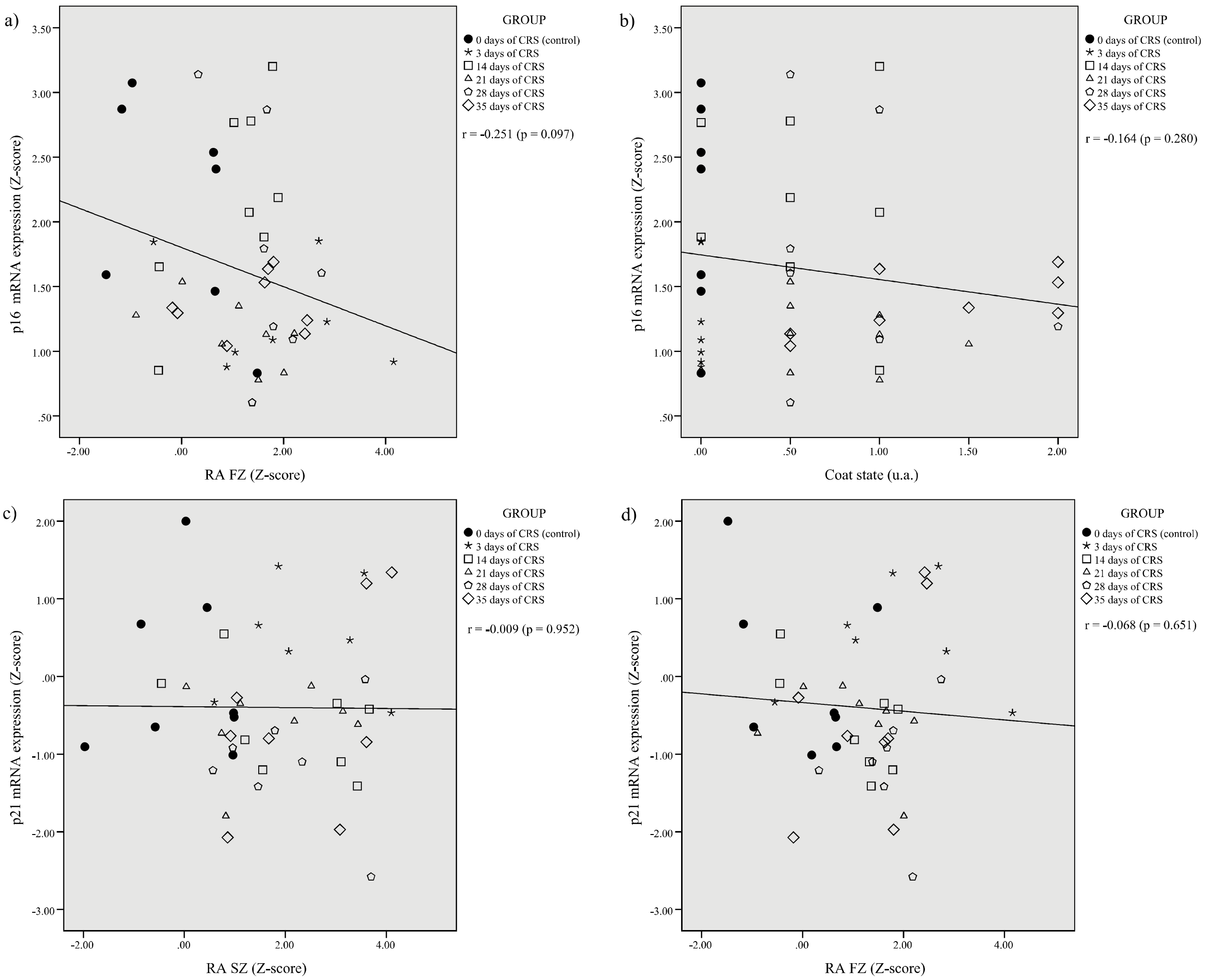

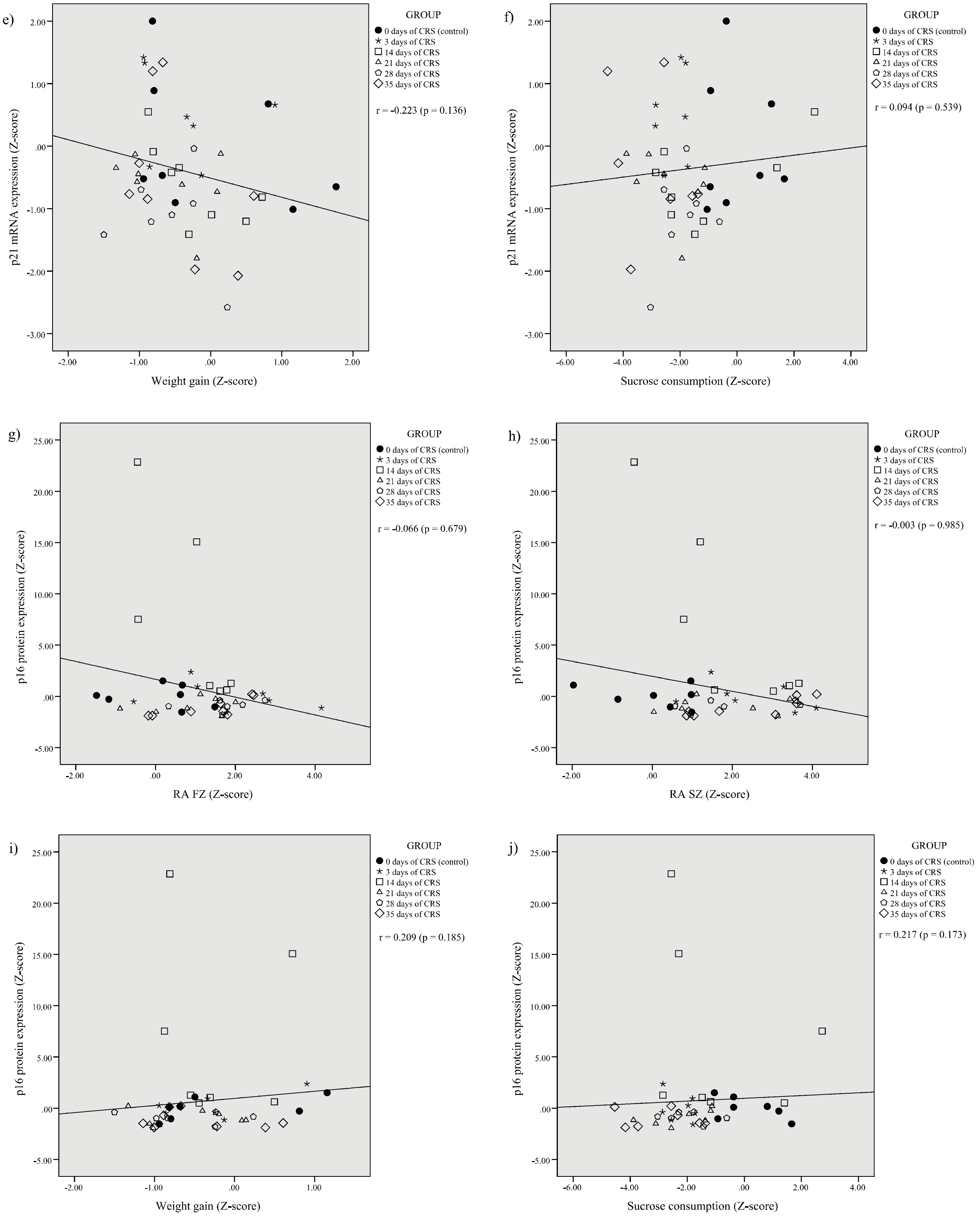
e)
p21 mRNA expression (Z-score)
Weight gain (Z-score)
GROUP
0 days of CRS (control)
3 days of CRS
14 days of CRS
21 days of CRS
28 days of CRS
35 days of CRS
r = -0.223 (p = 0.136)
f)
p21 mRNA expression (Z-score)
Sucrose consumption (Z-score)
r = 0.094 (p = 0.539)
g)
p16 protein expression (Z-score)
RA FZ (Z-score)
r = -0.066 (p = 0.679)
h)
p16 protein expression (Z-score)
RA SZ (Z-score)
r = -0.003 (p = 0.985)
i)
p16 protein expression (Z-score)
Weight gain (Z-score)
r = 0.209 (p = 0.185)
j)
p16 protein expression (Z-score)
Sucrose consumption (Z-score)
r = 0.217 (p = 0.173)

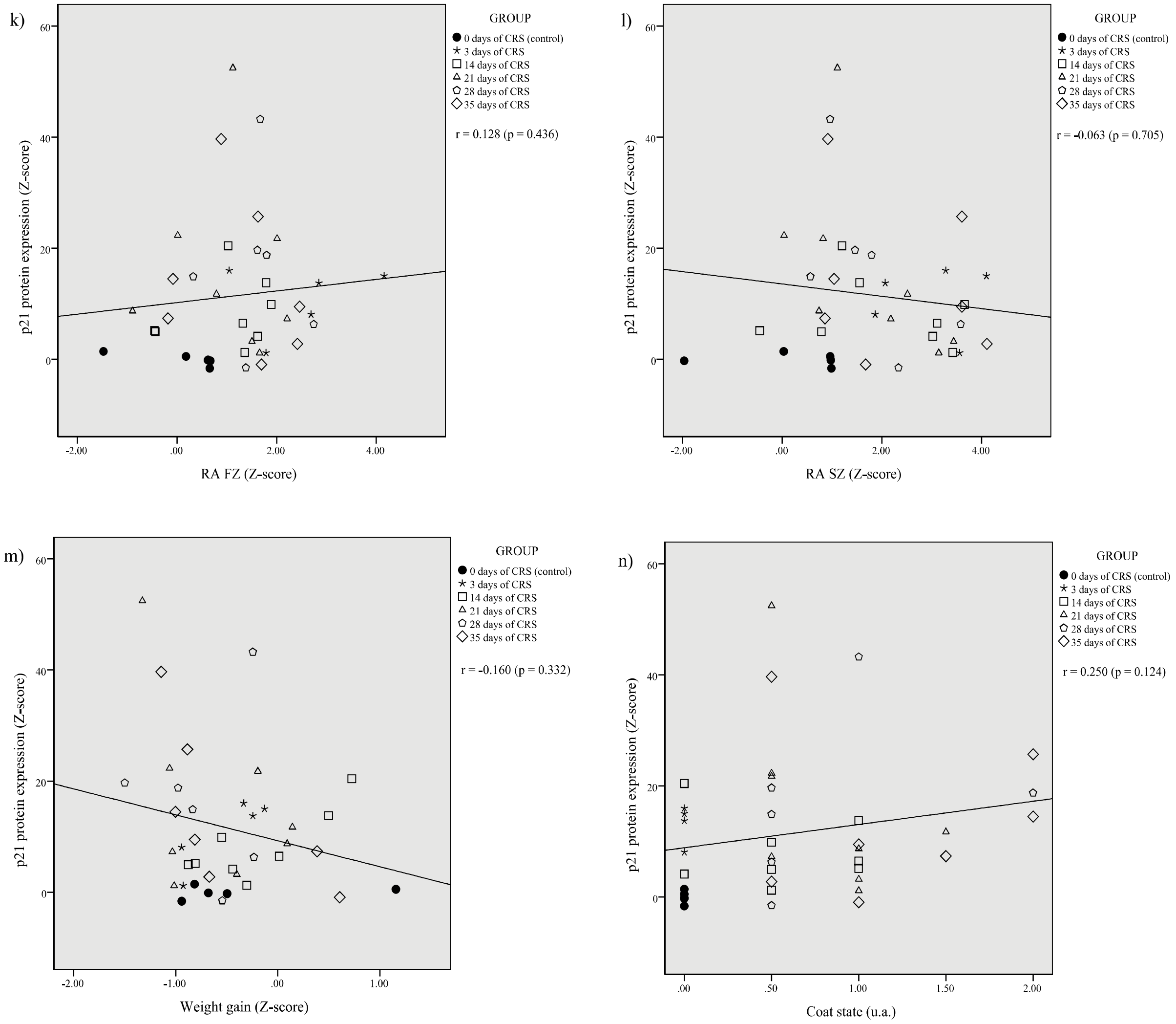
k)
GROUP
0 days of CRS (control)
3 days of CRS
14 days of CRS
21 days of CRS
28 days of CRS
35 days of CRS
r = 0.128 (p = 0.436)
p21 protein expression (Z-score)
RA FZ (Z-score)
l)
GROUP
0 days of CRS (control)
3 days of CRS
14 days of CRS
21 days of CRS
28 days of CRS
35 days of CRS
r = -0.063 (p = 0.705)
p21 protein expression (Z-score)
RA SZ (Z-score)
m)
GROUP
0 days of CRS (control)
3 days of CRS
14 days of CRS
21 days of CRS
28 days of CRS
35 days of CRS
r = -0.160 (p = 0.332)
p21 protein expression (Z-score)
Weight gain (Z-score)
n)
GROUP
0 days of CRS (control)
3 days of CRS
14 days of CRS
21 days of CRS
28 days of CRS
35 days of CRS
r = 0.250 (p = 0.124)
p21 protein expression (Z-score)
Coat state (u.a.)

1.3 Supplementary Figure 3 - Non-significant correlations results of cortical p16^{INK4a} and p21$^{Waf1/Cip1}$ expressions and behavioral performances in a sex-specific manner in mice submitted to Chronic restraint stress (CRS). Analysis revealed no significant correlations between p16^{INK4a} and p21$^{Waf1/Cip1}$ mRNA expression levels and residual avoidance in food zone (RA FZ) **(a and e, respectively)**, residual avoidance in shelter zone (RA SZ) **(b and f, respectively)**, and sucrose consumption **(c and g, respectively)** in both sexes. In addition, p16^{INK4a} mRNA expression levels were not correlated with coat state **(d)** in female or male. Protein levels of p16^{INK4a} and p21$^{Waf1/Cip1}$ were not correlated with weight gain **(h and k, respectively)** and residual avoidance in food zone (RA FZ) **(i and l, respectively)** in both sexes. In addition, p16^{INK4a} protein expression levels were not correlated with residual avoidance in shelter zone (RA SZ) **(j)**, and p21$^{Waf1/Cip1}$ protein levels were not correlated with coat state **(m)** in female or male

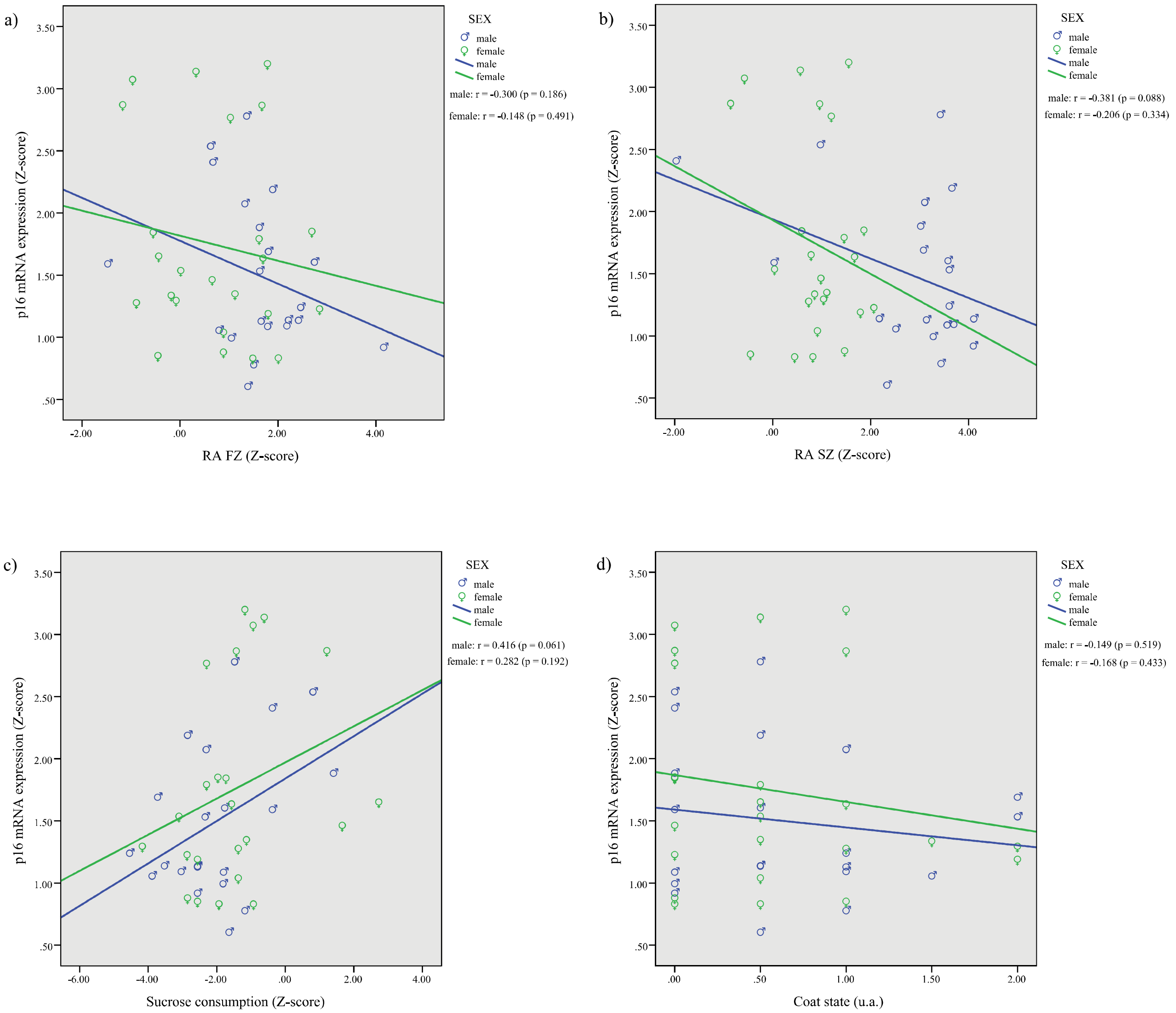

e)

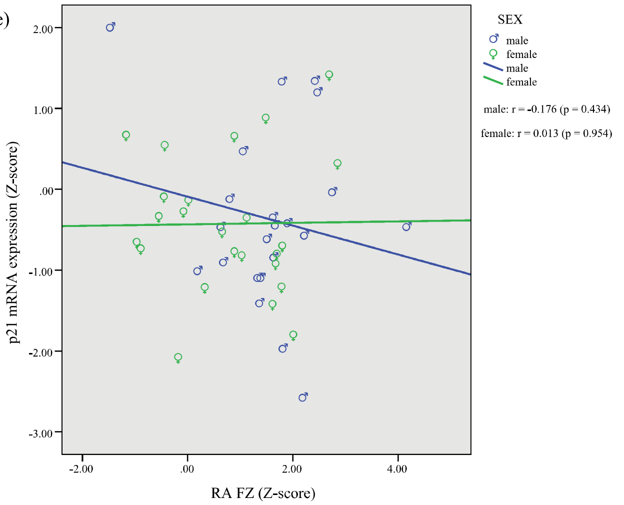
SEX
male
female
male
female
male: r = -0.176 (p = 0.434)
female: r = 0.013 (p = 0.954)
p21 mRNA expression (Z-score)
RA FZ (Z-score)

f)

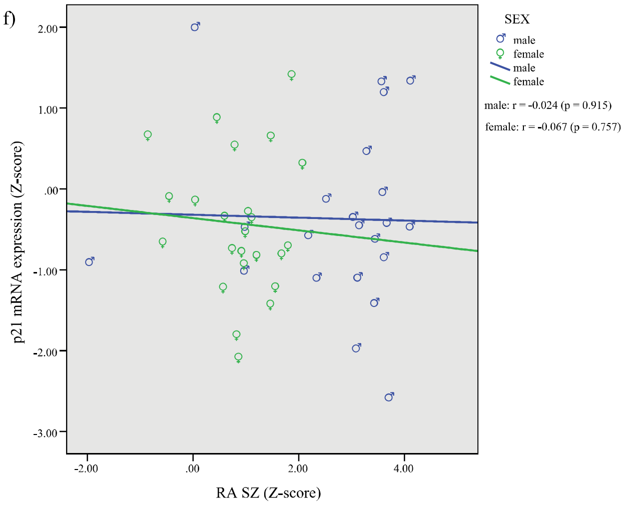
SEX
male
female
male
female
male: r = -0.024 (p = 0.915)
female: r = -0.067 (p = 0.757)
p21 mRNA expression (Z-score)
RA SZ (Z-score)

g)

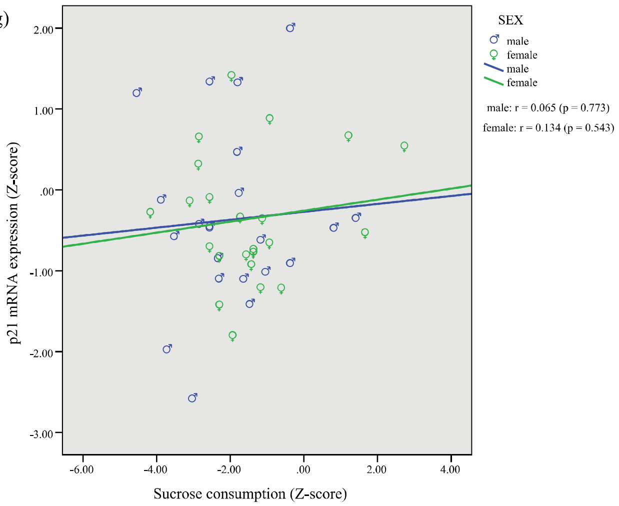
SEX
male
female
male
female
male: r = 0.065 (p = 0.773)
female: r = 0.134 (p = 0.543)
p21 mRNA expression (Z-score)
Sucrose consumption (Z-score)

h)

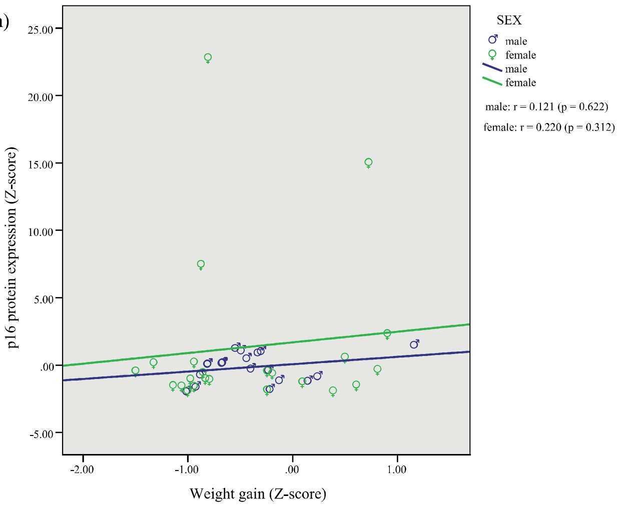
SEX
male
female
male
female
male: r = 0.121 (p = 0.622)
female: r = 0.220 (p = 0.312)
p16 protein expression (Z-score)
Weight gain (Z-score)

i)

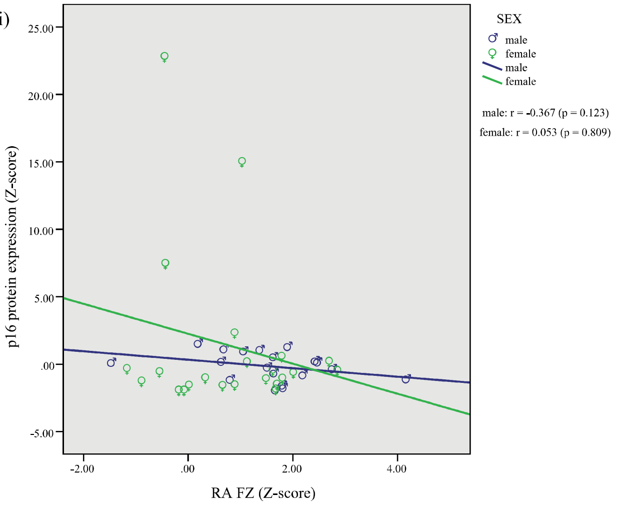
SEX
male
female
male
female
male: r = -0.367 (p = 0.123)
female: r = 0.053 (p = 0.809)
p16 protein expression (Z-score)
RA FZ (Z-score)

j)

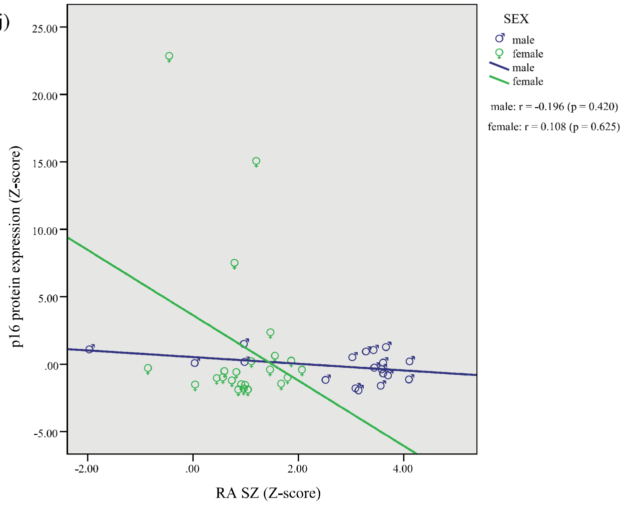
SEX
male
female
male
female
male: r = -0.196 (p = 0.420)
female: r = 0.108 (p = 0.625)
p16 protein expression (Z-score)
RA SZ (Z-score)

k)
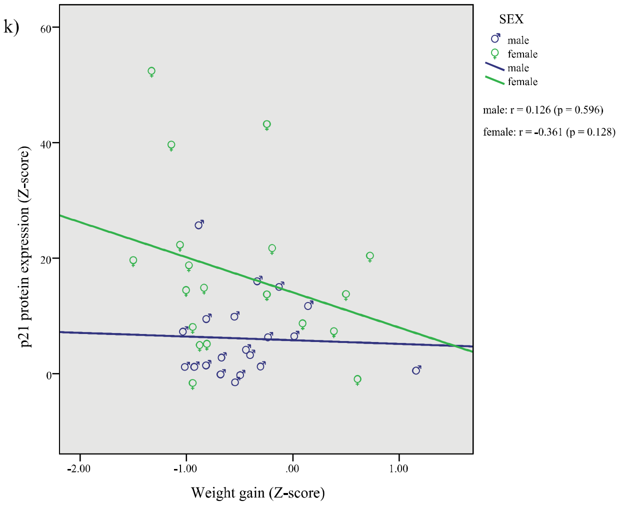
SEX
male
female
male
female
male: r = 0.126 (p = 0.596)
female: r = -0.361 (p = 0.128)
p21 protein expression (Z-score)
Weight gain (Z-score)

l)
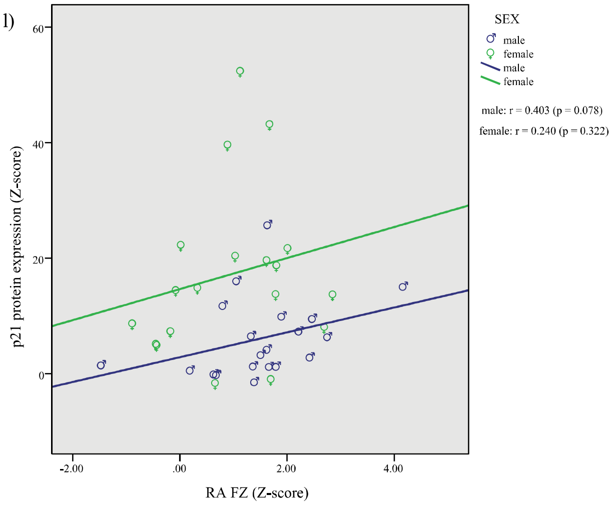
SEX
male
female
male
female
male: r = 0.403 (p = 0.078)
female: r = 0.240 (p = 0.322)
p21 protein expression (Z-score)
RA FZ (Z-score)

m)
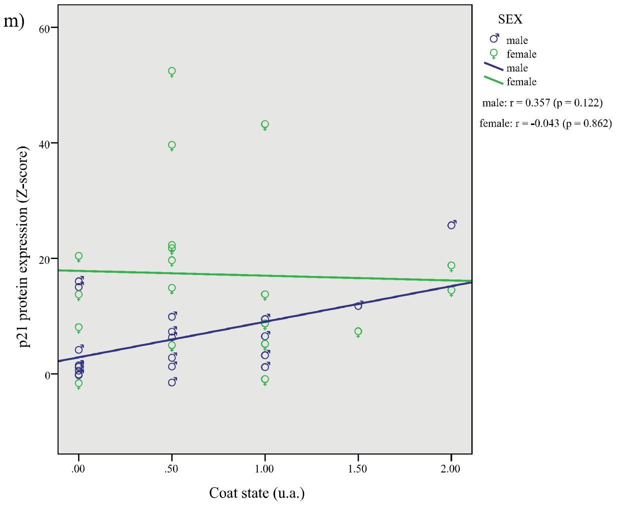
SEX
male
female
male
female
male: r = 0.357 (p = 0.122)
female: r = -0.043 (p = 0.862)
p21 protein expression (Z-score)
Coat state (u.a.)

1.4 Supplementary Figure 4 - A melting curve analysis was performed on samples using the BioRad CFX96 after RT-qPCR to confirm a single amplicon was produced for each candidate gene. Melt curve results including amplification, melt curve and melt peak plots for p16^{INK4a} **(a)**, p21$^{Waf1/Cip1}$ **(b)** and GAPDH **(c)** genes.

a)

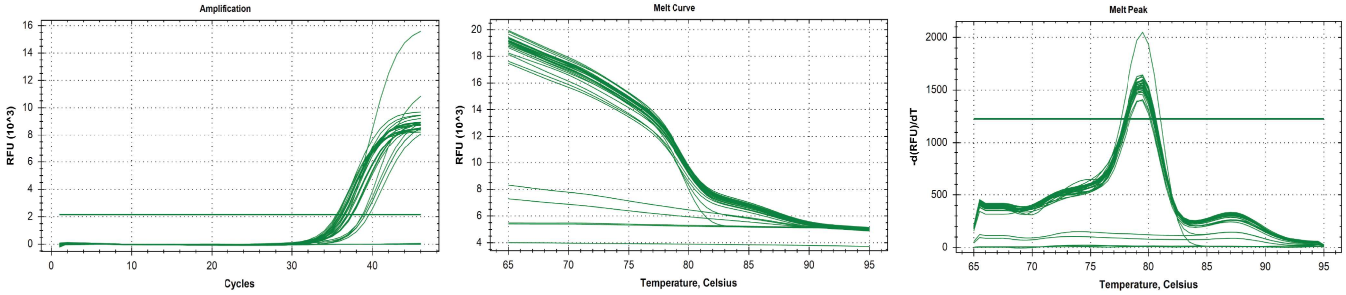

b)

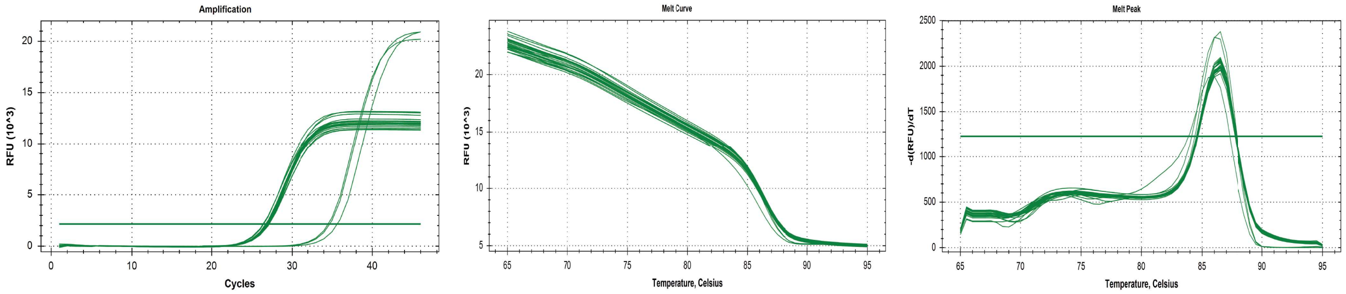

c)

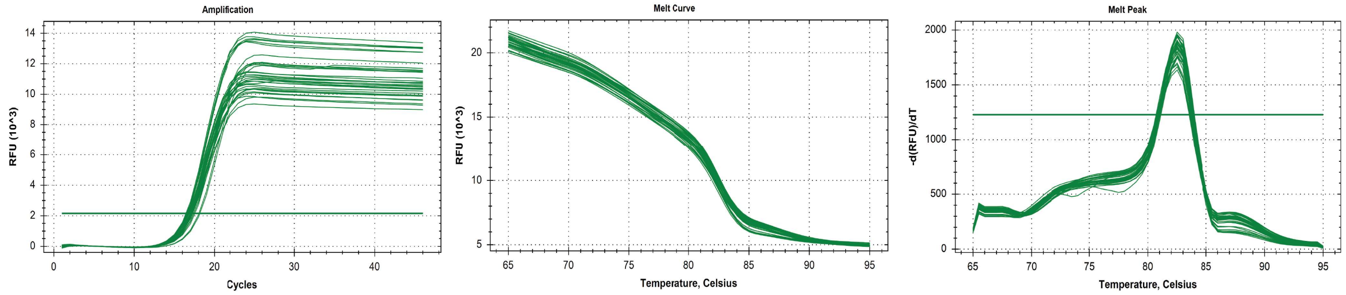

Supplement: Supplementary file 1 [file Data_Sheet_1.PDF]
